# The gut microbiota changed by ketogenic diets contribute to glucose intolerance rather than lipid accumulation

**DOI:** 10.3389/fendo.2024.1446287

**Published:** 2024-09-27

**Authors:** Wei Li, Mengxue Gong, Zhiyi Wang, Han Pan, Yue Li, Chenhong Zhang

**Affiliations:** State Key Laboratory of Microbial Metabolism, School of Life Sciences and Biotechnology, Shanghai Jiao Tong University, Shanghai, China

**Keywords:** ketogenic diet, gut microbiota, glucose and lipid metabolism, SCFA, bile acid

## Abstract

The ketogenic diet (KD) is a popular option for managing body weight, though its influence on glucose and lipid metabolism was still inconclusive. Gut microbiota is modulated by dietary pattens and has been associated with the changes of metabolic homeostasis induced by KD. Here, we found that two types of KDs, KD1 (8.8% carbohydrate, 73.4% fat, 17.9% protein, 5.7 kcal/g) and KD2 (0.4% carbohydrate, 93.2% fat, 6.4% protein, 6.7 kcal/g), induced changes of gut microbiota and its metabolites, contributing to glucose intolerance but not lipid accumulation in mice. Following a 2-week intervention with KDs, mice fed on KD1 displayed symptoms related to obesity, whereas KD2-fed mice exhibited a decrease in body weight but had severe hepatic lipid accumulation and abnormal fatty acid metabolism, while both KDs led to significant glucose intolerance. Compared to the mice fed on a standard chow diet, the conventional mice fed on both KD1 and KD2 had significant shifted gut microbiota, lower levels of short chain fatty acids (SCFAs) and composition alteration of cecal bile acids. By using an antibiotic cocktail (ABX) to deplete most of the gut microbiota in mice, we found the disturbances induced by KDs in lipid metabolism were similar in the ABX-treated mice to their conventional companions, but the disturbances in glucose metabolism were absent in the ABX-treated mice. In conclusion, these findings suggest that ketogenic diets disrupted glucose and lipid metabolism, at least in mice, and highlight the gut microbial culprits associated with KD induced glucose intolerance rather than lipid accumulation.

## 1 Introduction

The ketogenic diet (KD) is a high-fat, low-carbohydrate and moderate protein diet (1, 2). By severely restricting carbohydrates and increasing fat content, the ketogenic diet shifts the body’s primary energy source from glucose to ketone bodies (KB), which are intermediate products produced during fatty acid oxidation in the liver (3–6). Because of this, ketogenic diets are popular in weight loss management (7). Despite its effectiveness in promoting weight loss, numerous studies in both human and animal models have raised concerns regarding the adverse effects of KD on glucose and lipid metabolism. In human studies, consumption of KD has been associated with unfavorable alterations in lipid profiles, including elevated levels of LDL cholesterol and triglycerides (8). Additionally, KD interventions have been shown to induce transient increases in postprandial glucose levels, raising concerns about its impact on glucose metabolism (9). These findings are further supported by animal studies, which have demonstrated the development of hepatic insulin resistance induced by KD (10). However, the mechanism by which KDs influence glucose and lipid metabolism remains elusive.

Previous studies have highlighted the potential impact of the ketogenic diet (KD) on gut microbiota composition and bacterial metabolites (11–14). A systematic review reveals that KD can lead to a decrease in the abundance of *Bifidobacterium* and butyrate-producing bacteria belonging in Firmicutes, resulting in lowered levels of SCFAs, potentially promoting obesity, gastrointestinal disorders, and T2D (15). Our previous research also showed significant alterations in the composition and content of SCFAs, bile acids, and tryptophan metabolites in mice fed with KDs, which were closely related to the disorders of glucose and lipid metabolism (16). Despite these observations, our understanding of the mechanistic pathways by which gut microbiota and microbe-associated metabolites influence the metabolic effects of KD remains limited. Therefore, further investigation is warranted to elucidate the complex interplay between KD, gut microbiota, and host metabolism.

In this study, we aimed to investigate the impact of ketogenic diets (KDs) on glucose and lipid metabolism and elucidate the role of gut microbiota in the metabolic disturbances induced by KDs. To achieve this, we employed two types of KDs: KD1, typically used in clinical trials (7), and KD2, commonly used in mice trials (17). Through short-term dietary interventions in both specific pathogen-free (SPF) mice and microbiota-depleted mice, we sought to explore the contribution of gut microbiota to KD-induced metabolic alterations. Our findings revealed that both KDs induced glucose and lipid metabolism disorders, but only glucose intolerance induced by KDs depended on gut microbiota. This study provides future research directions for exploring the mechanisms underlying the effects of KD on host glucose and lipid metabolism, and it emphasizes the critical role of gut microbiota in the mechanisms of KD.

## 2 Materials and methods

### 2.1 Animal trials

Five-week-old male C57BL/6 mice were obtained from SLAC Inc. (Shanghai, China) and then kept under the specific pathogen-free (SPF) conditions at the animal facility of Shanghai Jiao Tong University, Shanghai, China (12h light/dark phase cycle, temperature at 22°C ± 3°C). All animal experimental procedures were approved by the Institutional Animal Care and Use Committee (IACUC) of Shanghai Jiao Tong University (No. A2021043).

#### 2.1.1 Trial 1

After one-week of acclimation, the mice were provided normal chow (NC, 3.9 kcal/g, with 65.6% of calories from carbohydrate, 16.3% from fat, and 18% from protein) and pure drinking water for 4 weeks. Then the mice were randomly assigned to 3 groups: (1) NC group (n = 10), control mice fed normal chow; (2) KD1 group (n = 10), mice fed a ketogenic diet commonly used in clinical studies (KD1, 5.7 kcal/g, with 8.8% of calories from carbohydrate, 73.4% from fat, and 17.9% from protein); (3) KD2 group (n = 10), mice fed a ketogenic diet commonly used in animal trials (KD2, 6.7 kcal/g, with 0.4% of calories from carbohydrate, 93.2% from fat, 6.4% from protein). All mice were allowed ad libitum access to water and freshly prepared food every day for 2 weeks. The body weight of the mice was measured every day.

#### 2.1.2 Trial 2

After one-week of acclimation, a cocktail of antibiotics (0.5 g/L of vancomycin, 1 g/L of ampicillin, 1 g/L of neomycin, 1 g/L of metronidazole) was introduced in drinking water for 4 weeks. The mice were then randomly assigned to 3 groups: (1) ABX + NC group (n = 10), fed normal chow (NC). (2) ABX + KD1 group (n = 10), fed KD1. (3) ABX + KD2 group (n=10), fed KD2. The mice were allowed ad libitum access to water and food for 2 weeks. During the 2 weeks, all groups continued to have the antibiotic cocktail in their drinking water. All mice were fed freshly prepared diets every day and the body weight were measured every day.

Both trials used the same batch of mice and started at the same time. All diets involved were produced by SYSE Ltd., Changzhou, China. The formulas of these diets are shown in **Supplementary Table S1**. Fecal samples were collected at week 2 and stored at -80°C for gut microbiota analysis. All mice were fasted for 6 h before sampling. Blood samples were collected from the orbital vascular plexus. Serum samples were isolated from blood samples after centrifugation for 15 min at 4°C at 3,000 g and then stored at -80°C. Liver, pancreas, adipose tissues (epididymal, retroperitoneal, perirenal, mesenteric), cecum contents and colon contents were weighed and collected immediately and stored at -80°C or in 4% paraformaldehyde for further analysis.

### 2.2 Oral glucose tolerance test

All mice were fasted for 6 h and then administered glucose (2 g/kg body weight) through oral gavage. The blood glucose concentrations were measured from the tip of the tail vein at 0 (the fasting glucose) and 15, 30, 60, 90, and 120 min after glucose administration using a blood glucose meter (Accu-Chek Performa, Roche, USA). Blood samples were collected from the tail vein into tubes at 0, 15, and 60 min after glucose administration.

### 2.3 Serum insulin measurement

Enzyme-linked immunosorbent assays (ELISAs) were used to measure the serum insulin (90080, Chrystal Chem, USA) in accordance with the manufacturer’s instructions.

### 2.4 Blood ketone measurement

After 6 h of fasting, blood ketone (β-hydroxybutyrate) levels were measured in blood samples collected from the tip of the tail vein with a blood ketone meter (FreeStyle Optium Neo, Abbott, USA).

### 2.5 Liver triglycerides measurement

Frozen liver samples were homogenized in a corresponding volume (w:v = 1:9) of absolute ethanol. The supernatant was collected after centrifugation for 25 min at 2,000×g and 4°C. Triglycerides in the homogenized tissue were quantified using colorimetric kits from Nanjing Jiancheng Bioengineering Institute (Nanjing, China).

### 2.6 Serum ALT and AST measurement

The concentrations of ALT and AST in serum were quantified with kits (C009-2-1 and C010-2-1, respectively) from Nanjing Jiancheng Bioengineering Institute (Nanjing, China).

### 2.7 Histopathology of epididymal fat, perirenal fat, liver and pancreas

For epididymal fat and liver, the fresh epididymal fat pad and the largest lobe of the liver were fixed with 4% paraformaldehyde solution for 48 hours. After the tissues were embedded in paraffin, the sections about 4 μm thickness were stained with hematoxylin and eosin (H&E). The size of adipocytes and the area of liver steatosis were calculated using Image-Pro Plus v6.0 (Media. Cybernetics Inc., Silver Springs, MD). The specific steps were as follows: for adipose tissue, the number of adipocytes in three pictures (400× magnification) was counted for each sample, and the average adipocyte size was calculated based on the total area (cross-sectional area) divided by the number of adipocytes. For liver tissue, a histological score (NAFLD Score) was performed according to the method described by David E et al. (18).

For perirenal adipose tissue, the right fat pad was directly frozen in liquid nitrogen during dissection, and then stored at -80°C. The frozen tissue was subsequently dehydrated, embedded in paraffin, sectioned, and stained with hematoxylin and eosin (H&E) (Wuhan Seville Biotechnology Co., Ltd). The size of adipocytes was then calculated using Image-Pro Plus v6.0 (Media. Cybernetics Inc., Silver Springs, MD).

For pancreatic tissues, fresh tissues were fixed with 4% paraformaldehyde for 48 h, embedded in paraffin, sectioned, and stained with hematoxylin and eosin (H&E). Subsequently, a panoramic scan of the sections was conducted using 3DHISTECH Pannoramic. The number of islets in each section was counted using Case Viewer v2.4. In addition, the embedded wax blocks of mouse pancreatic tissue were sliced into sections approximately 4 μm thick and stained for insulin after standard indirect immunofluorescence staining. Finally, Image Pro Plus v6.0 (Media. Cybernetics Inc., Silver Springs, MD) was used to calculate the insulin positive area and the total area in the scanned images of each section, and divided the insulin positive area by the total area to obtain the percentage of insulin positive area.

### 2.8 Quantification of mRNA expression in epididymal fat, liver and pancreas

The total RNA was extracted using RNA extraction kits (RNeasy Plus Universal Tissue Mini Kit, 73404, Qiagen) according to manufacturer’s instruction. The concentration of the total RNA was determined by agarose gel electrophoresis and NanoPhotometer (NP80, IMPLEN). Then the RNA was reverse transcribed into cDNA according to the instructions of the kit (SuperScriptTM III First-Str Synthesis, 18080051, Invitrogen). Then the cDNA was amplified in a 20 μL reaction system to detect the expression levels of related genes (**Table 1**) by qPCR with the instrument (qTOWER3G, Analytik Jena). The SYBR Green (IQ SYBR Green Supermix, 170-8882AP BIO-RAD) was used as fluorescence chromogenic system and the primer sequences of related genes are as listed in **Table 1**. The PCR conditions were 95°C for 3 min, followed by 40 cycles of 95°C for 20 s, 56°C for 30 s, and 72°C for 30 s, and plate reads for 5 s. Gene expression levels were determined using the comparative ΔΔC_T_ method (2^-ΔΔCT^ method).

**Table 1:** Related genes and the primer sequences for qPCR.

Gene	Primer-F	Primer-R
*Fatp1*	GGAAGAGCCTCCTCAAGTTCT	GGTCCAGGAGCTGCGTGTCA
*Cd36*	AGATGACGTGGCAAAGAACAG	CCTTGGCTAGATAACGAACTCTG
*Acsl1*	GGAGGACCTTGGAAGAGTGAA	ATCTGTGGAAGCGATGAATGC
*Cpt1a*	AGATCAATCGGACCCTAGACAC	CAGCGAGTAGCGCATAGTCA
*Dgat2*	GTGCACAAGTGGTGCATCA	CAGTGGGACCTGAGCCATC
*Atgl*	GCCAACGCCACTCACATCTACG	GACAGCCACGGATGGTCTTCAC
*Hsl*	ACCATCAACCGACCAGGAGTGCTCTT	GCCCGTCTCGTTGCGTTTGTAGTGT
*GAPDH*	GACCCCTTCATTGACCTCAAC	CGCTCCTGGAAGATGGTGAT
*G6pase*	CGAGGAAAGAAAAAGCCAAC	CAAGGTAGATCCGGGACAGA
*Pck1*	TGTCGGAAGAGGACTTTGAGA	CCACATAGGGCGAGTCTGTC
*Pdx1*	CCTTTCCCGAATGGAACCGA	GGGCCGGGAGATGTATTTGT
*Ins1*	TTCTACACACCCAAGTCCCG	AAGTTTTATTCATTGCAGAGGGGTG
*Glut2*	CAGTCACACCAGCATACACAA	TGATACACTTCGTCCAGCAATG
*β-actin*	AAGACCTCTATGCCAACACAGT	CTGCTTGCTGATCCACATCTG

### 2.9 Fecal DNA isolation

Fecal DNAs of mice at week 2 (Trial 1) were isolated according to the method described by Goden et al. (19). The concentration of DNA was measured by NanoPhotometer (NP80, IMPLEN). All DNA samples were diluted to a concentration of 10 ng/μL and stored at -20°C.

### 2.10 Sequencing of 16s rRNA gene V3-V4 region of gut microbiota and data analysis

The sequencing library of 16S rRNA gene V3-V4 region was conducted following the sequencing guidelines of Illumina with improvement as previously described (20). And sequenced on the Illumina MiSeq platform (Illumina, Inc., San Diego, CA, USA). The raw data were analyzed in QIIME2(Quantitative Insights Into Microbial Ecology, Version 2021.4). The main steps are as follows: import the raw sequences, use the “Cutadapt” plug-in to remove the connectors and primers, and DADA2 to prune the sequence (retain the first 269 bp of all forward sequences and the first 181 bp of reverse sequences). The sequence information and the original abundance file of amplified subsequence variant (ASVs) were obtained after filtering, denoising, de-chimerism and merging. All samples were rarefied to 24000 reads to normalize sequencing depth and calculate the relative abundance, then the phylogenetic tree was constructed using the “FastTree” plug-in based on the sequence information of ASVs. Taxonomic classification of ASVs was performed using the SILVA132 database.

The alpha diversity of the samples was evaluated using the observed ASVs and Shannon index. Principal coordinate analysis (PCoA) based on Bray-Curtis distance was carried out using the relative abundance matrix of ASVs to evaluate the beta diversity. The statistical significance of gut microbiota among different groups was assessed by permutational multivariate analysis of variance test (PERMANOVA) with 9999 permutations.

Redundancy analysis (RDA) was carried out following the method described by Forester et al. (21). The analysis and plotting were carried out using R (v4.1.3). Firstly, the relative abundance matrix of ASVs was standardized using Hellinger transformation, and the actual intake of carbohydrates, fats and proteins in each group of mice served as environmental variables. Subsequently, the environmental variables were transformed by log1p, the “psych” package was used to analyze the environmental factors, excluding those with a variance inflation factor greater than 10. Redundancy analysis of the microbiota structure was then carried out, with carbohydrate intake and protein intake, or fat intake and protein intake, as environmental variables. The Mantel test was used to analyze the degree of explanation of each RDA axis to the variation of the overall microbiota structure and evaluate the significance. The ASVs related to different environmental factors and significantly affecting the overall microbiota structure were selected based on specific criteria: in the RDA triple sequence diagram, the projection value of the angle with the direction of the environmental factor exceeded 0.6, and the distribution on the RDA1 or RDA2 axis was more than 1.96 standard deviations (p < 0.05). The relative abundance of ASVs was visualized using the ‘pheatmap’ package in R, and differences in the relative abundance of each ASV across different groups were analyzed using the Mann-Whitney U test in GrapadPrism v8.0.

Based on the sequence information of ASVs, the functional genes of the representative strains of ASVs were predicted by PiCRUSt2 (22). Combined with the ASV relative abundance matrix, the relative abundance of functional genes and metabolic pathways in the samples was obtained and annotated based on the KEGG database. Subsequently, the data were visualized using an online platform developed by Chen Tong et al. (23).

### 2.11 Short−chain fatty acids profiling

To determine the SCFAs in cecum content, all operations were conducted on ice to minimize volatilization. For the standards: accurately measure 400 μL of acetic acid, 200 μL of propionic acid, 200 μL of butyric acid, 20 μL of isobutyric acid, 30 μL of isovaleric acid, and 40 μL of valeric acid, then add water to make a total volume of 5 mL. The standard curve was prepared by diluting 2 mL of the masterbatch to 20 mL, and then further diluted in a two-fold gradient. 200 μL of each gradient standard was used for subsequent acidification, extraction and detection. For the cecal contents, phosphate buffer solution (PBS, 0.01 M) was added (w/v = 1:5), fully mixed and homogenized by Tissuelysser II (QIAGEN, Germany). The mixture was then centrifuged at 16,000 g for 15 minutes at 4°C, and the supernatant was collected and transferred to a new 1.5 mL tube, then filtered through a 0.22 μm aseptic membrane to obtain the fecal water. Next, 200 μL of fecal water of each sample was acidified with 100 μL of 50% (v/v) sulfuric acid. This mixture was then combined with 400 μL of anhydrous ether, followed by 2 min of static incubation on ice for extraction. The supernatant was collected by centrifugation at 12,000 g for 5 minutes at 4°C. Finally, the concentration of short-chain fatty acids (SCFAs) was measured by gas chromatography (Agilent 6890, Agilent Technologies, USA), using a DB-FFAP (0.25 mm × 30 m × 0.25 μm) column and hydrogen flame ionization.

### 2.12 Bile acids profiling

The concentrations of bile acids in colon content were determined by liquid chromatography-mass spectrometry (LC-MS). The operations were conducted by Suzhou Panomick Biotechnology company, using an EXionLC liquid chromatograph (EXionLC, ABSCIEX) and mass spectrometer (AB6500Plus, ABSCIEX). Approximately 50 mg of colon content of each sample (n=9 for each group) was used for detection.

### 2.13 Statistical analysis

In the measurement of physiological index and short-chain fatty acid concentrations, samples from all 10 mice in each group were used. For the measurement of bile acid concentrations, samples from 9 mice in each group were used. Statistical analysis and plots were performed using GraphPad Prism (version8.0). Firstly, ROUT (Q = 1%) was used to test each group of data and eliminate outliers. One-way ANOVA and Tukey’s multiple comparison test were used to analyze the differences between groups. The Mann-witney U test was used to compare the relative abundance of intestinal microorganisms between groups. P values < 0.05 were considered statistically significant.

## 3 Results

### 3.1 Ketogenic diets induced disorders in both glucose and lipid metabolism in SPF mice

To explore the effects of two distinct ketogenic diets on fat and glucose metabolism in mice, SPF C57BL/6J mice were divided into three groups and fed the specific diets: normal chow diet (NC, comprising 65.6% carbohydrate, 16.3% fat, and 18.0% protein), ketogenic diet 1 (KD1, comprising 8.8% carbohydrate, 73.4% fat, and 17.9% protein, a ketogenic diet commonly employed in clinical studies), or ketogenic diet 2 (KD2, comprising 0.4% carbohydrate, 93.2% fat, and 6.4% protein, a ketogenic diet commonly employed in animal trials). Formulas are provided in **Supplementary Table S1**. After two weeks of feeding, the fasting blood ketone levels in both KD1 and KD2 groups were significantly elevated compared to the NC group (**Supplementary Figure S1A**), with KD2 mice exhibiting higher blood ketone level than KD1 mice.

**Figure 1:**
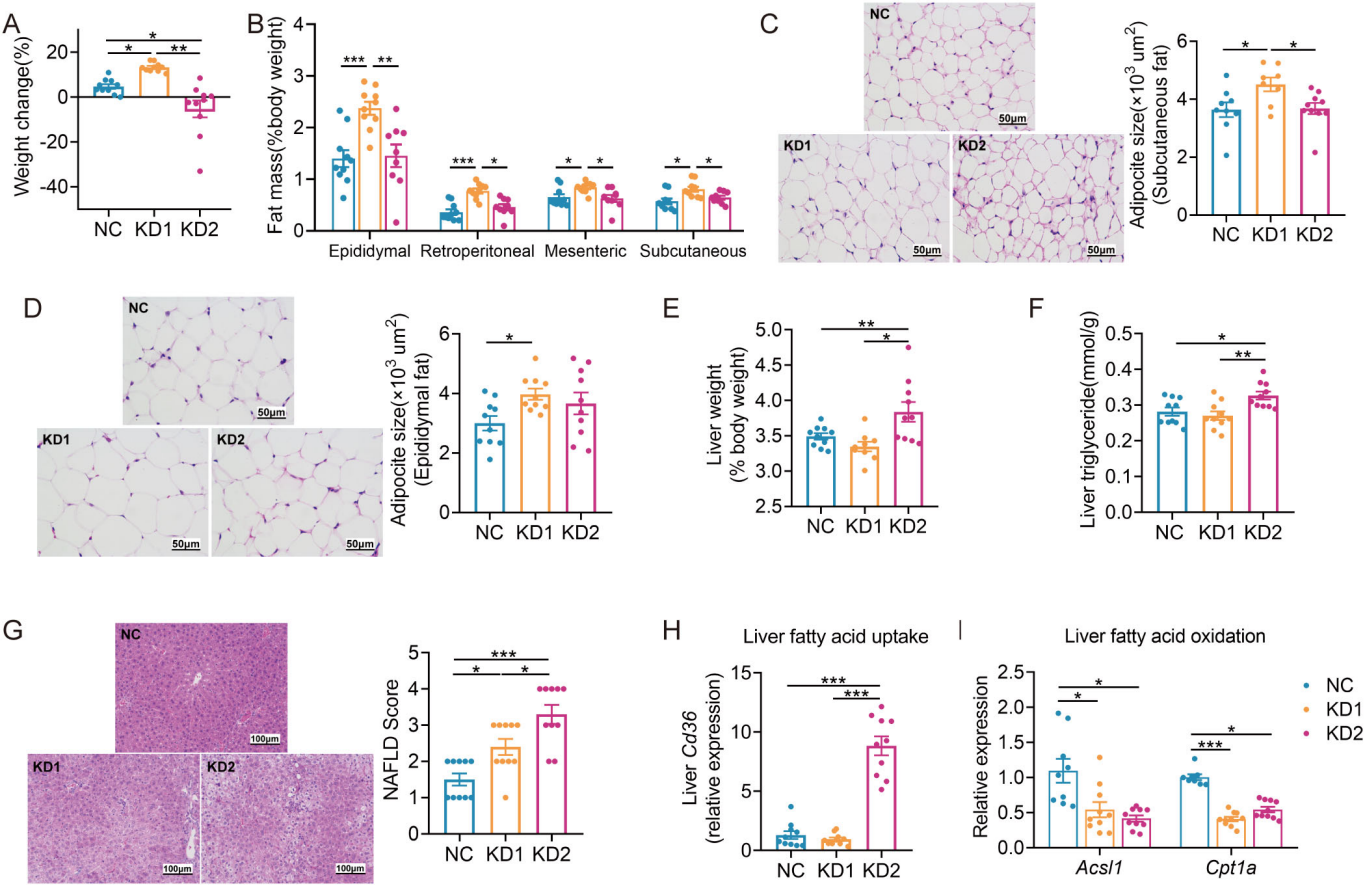
KDs induced lipid accumulation in mice. **(A)** Changes of body weight. **(B)** Weight of adipose tissue (epididymal fat, perirenal fat, mesenteric fat and subcutaneous fat, %body weight). **(C, D)** Representative H&E-stained histological sections of subcutaneous fat and epididymal fat (scale: 50 µm) and calculated cell size of adipocytes. **(E)** Liver weight. **(F)** Concentration of liver triglyceride. **(G)** Representative H&E-stained histological sections of liver (scale: 100 µm) and calculated histologic score (NAFLD Score). **(H, I)** Relative mRNA expression of genes involved in **(H)** fatty acid uptake (*Cd36*) and **(I)** oxidation (*Acsl1*, *Cpt1a*) in liver. Data were presented as mean ± SEM and analyzed using one-way ANOVA test, followed by Tukey’s multiple comparisons test. ROUT (Q = 1%) was used in each group to eliminate outliers. *p < 0.05, **p < 0.01, ***p < 0.001, n = 10 for each group.

Compared to the NC mice, KD1 mice exhibited increased body weight, while KD2 mice decreased significantly throughout the dietary intervention (**Figure 1A**, **Supplementary Figure S1B**). Additionally, fat pad weight and adipocyte size significantly increased in the KD1 group compared to the NC group, while there was no significant difference between the KD2 and NC groups (**Figures 1B–D**). Furthermore, only the KD2 group showed an increase in liver weight and triglyceride content (**Figures 1E, F**). Both the KD1 and KD2 groups had significantly higher non-alcoholic fatty liver (NAFLD) scores (**Figure 1G**). KD2 mice also had significantly higher serum ALT levels compared to the NC mice (**Supplementary Figures S1C, D**). These findings suggest that both KD1 and KD2 induced lipid accumulation in mice, with an increase in fat pad of KD1 mice but a significant increase in liver observed in KD2 mice.

To probe the causes of lipid accumulation induced by these two KDs, we determined the expression levels of several genes associated with fatty acid oxidation, lipid droplet breakdown, and increased fatty acid uptake in the liver and epididymal fat. Interestingly, we found that the fatty acid uptake related gene *Cd36* was significantly upregulated only in the KD2 group compared to the NC group, while both KD1 and KD2 downregulated the fatty acid oxidation related gene *Acsl1* in the liver (**Figures 1H, I**). Additionally, in the epididymal fat of KD1 mice, we observed significant downregulation of *Acsl1* and the lipid droplet breakdown-related gene *Hsl* (**Supplementary Figures S1F, H**). In contrast, KD2 mice exhibited upregulation of the fatty acid oxidation-related gene *Cpt1a* compared to the NC mice (**Supplementary Figure S1F**). These findings suggest that the KD disrupts the balance between fatty acid oxidation and uptake in the liver and epididymal fat of mice, contributing to disorders in lipid metabolism.

**Figure 2:**
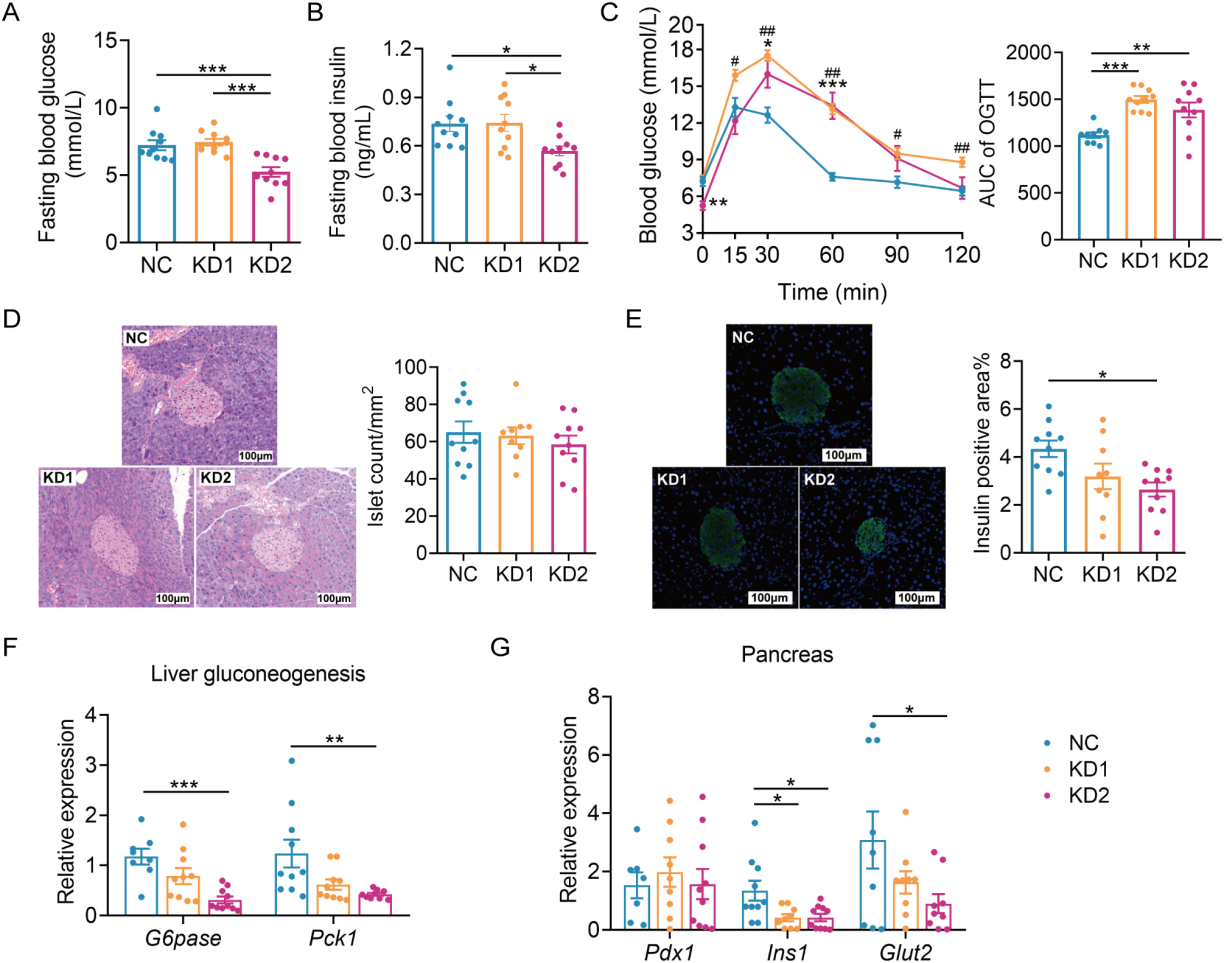
KDs induced glucose intolerance in mice. **(A)** Fasting blood glucose. **(B)** Fasting serum insulin. **(C)**Blood glucose curves and areas under the curve (AUC) during the oral glucose tolerance test (OGTT). **(D)** Representative H&E-stained histological sections of pancreas and calculated islet number. **(E)** Representative insulin immunofluorescence-stained (green) histological sections of pancreas (scale bar = 100 μm) and calculated mean insulin-positive area. **(F)** Relative mRNA expression of genes involved in liver gluconeogenesis. **(G)** Relative mRNA expression of genes of pancreas. All data were presented as mean ± SEM and analyzed using one-way ANOVA test, followed by Tukey’s multiple comparisons test among NC, KD1 and KD2 groups. In **(C)**, # and * represent the results of Tukey’s multiple comparison test between KD1 group and NC group, KD2 group and NC group respectively. ROUT (Q = 1%) was used in each group to eliminate outliers. *p < 0.05, **p < 0.01, ***p < 0.001, ^#^ *p* < 0.05, ^##^ *p* < 0.01, n = 10 for each group.

We then assessed the impact of these two types of KD on glucose metabolism. We conducted an oral glucose tolerance test (OGTT) after 2 weeks of dietary intervention and found that KD2 mice exhibited lower fasting blood glucose and insulin levels compared to NC mice, while no significant differences were observed between the KD1 and NC groups (**Figures 2A, B**). Moreover, both KD1 and KD2 mice showed slower glucose clearance compared to NC mice (**Figure 2C**). Although the number of islets were similar across all three groups (**Figure 2D**), the insulin positive area was significantly reduced in the KD2 group compared to the NC group, with no notable difference observed between the KD1 and NC groups (**Figure 2E**). Interestingly, gluconeogenesis related genes *G6pase* and *Pck1* were significantly decreased in the liver of KD2 mice but not in KD1 mice (**Figure 2F**). Additionally, both KD1 and KD2 groups exhibited significantly decreased expression of pancreas *Ins1*, with *Glut2* downregulated in the KD2 group. However, expression of *Pdx1*, a gene associated with pancreatic development, did not significantly differ among the three groups (**Figure 2G**). Overall, these findings indicate that both KD1 and KD2 induced glucose intolerance, likely attributable to insulin synthesis.

### 3.2 Ketogenic diets changed gut microbiota structure and bacterial metabolites in mice

**Figure 3:**
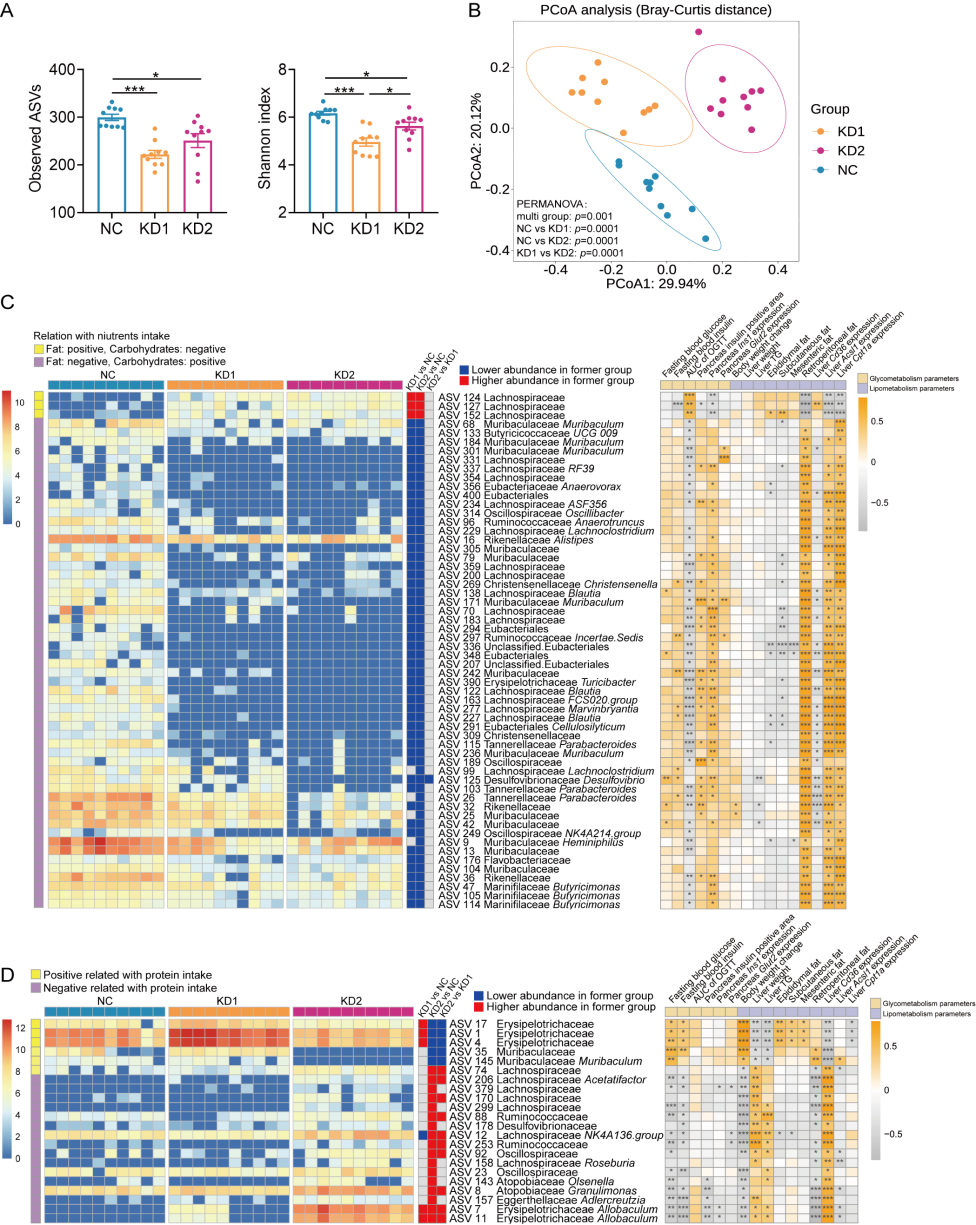
KDs changed the structure of gut microbiota. **(A)** Observed ASVs. and Shannon Index. **(B)** Principal coordinate analysis (PCoA) plot of gut microbiota based on Bray-Curtis distance. 95% confidence ellipses were added in different groups, and Permutational Multivariate Analysis of Variance (PERMANOVA) under 9999 permutations was used for microbial structure comparation. **(C, D)** Heatmap of selected ASVs responding to carbohydrates/fat **(C)** and protein **(D)** intake. Left: The heatmap shows the relative abundance (log-transformed) of ASVs in each sample, clustered by ward.D method. Medium: The changing direction of 58 ASVs through pairwise comparison of groups analyzed by the two-tailed tested using Mann-witney test, *p* < 0.05 was considered significant. The blue indicated that the former was significantly lower than the latter, red indicated that the former was significantly higher than the latter and gray indicated that there was no significant difference between the two groups. Right: Spearman correlations between the relative abundance of selected ASVs and the host parameters related to metabolism. Positive correlation (yellow), negative correlation (gray). *p* values were FDR corrected, *p < 0.05, **p < 0.01, ***p < 0.001. n = 10 for each group.

Diet is one of the important factors affecting the structure and function of gut microbiota. To explore the effects of the two types of KD on the gut microbiota of mice, we conducted sequencing of the 16S rRNA gene V3-V4 region of fecal samples collected post dietary intervention. The richness and diversity of gut microbiota, as reflected by the numbers of observed amplicon sequence variants (ASVs) and the Shannon index, were significantly decreased in KD1 and KD2 mice compared to the NC mice (**Figure 3A**). Principal-coordinate analysis (PCoA) and permutational multivariate analysis of variance (PERMANOVA) based on Bray-Curtis distance showed that there were significant differences in microbiota structure among the three groups (**Figure 3B**). In short, both KD1 and KD2 change the gut microbiota structure of mice.

To explore how gut microbiota contributes to glucose and lipid metabolism disorders induced by ketogenic diets (KDs), we used redundancy analysis (RDA) to identify specific bacterial members responsive to these dietary types. We selected 58 ASVs associated with carbohydrate and fat intake, and 22 ASVs linked to protein intake, based on nutrient consumption in each group (**Supplementary Table S2**, **Supplementary Figure S2**). Among the ASVs related to carbohydrate and fat intake, 50 ASVs showed significant correlations with parameters of glucose intolerance (**Figure 3C**). Notably, three ASVs from the Lachnospiraceae family (ASV124, ASV127, ASV152) were notably enriched, positively correlating with OGTT AUC and negatively correlating with *Ins1* mRNA expression (**Figure 3C**). Regarding lipid metabolism, a few ASVs correlated significantly with changes in body weight, liver weight, and mesenteric fat, while nearly all 58 ASVs showed positive correlations with liver fatty acid oxidation gene expression (**Figure 3C**). Furthermore, 55 ASVs were significantly downregulated by both KD1 and KD2 interventions. As for the 22 ASVs related to protein intake, KD2 significantly altered the abundance of all these ASVs (**Figure 3D**). Among them, 18 ASVs correlated with fasting blood glucose levels and 21 ASVs correlated with changes in body weight (**Figure 3D**). Additionally, Erysipelotrichaceae (ASV17, ASV1, ASV4) increased significantly in KD1 mice but decreased in KD2 mice, correlating positively with fasting glucose and insulin levels. In summary, the carbohydrate/fat and protein composition of KDs influenced specific gut bacteria in mice, which in turn correlated with parameters of glucose and lipid metabolism disorders.

**Figure 4:**
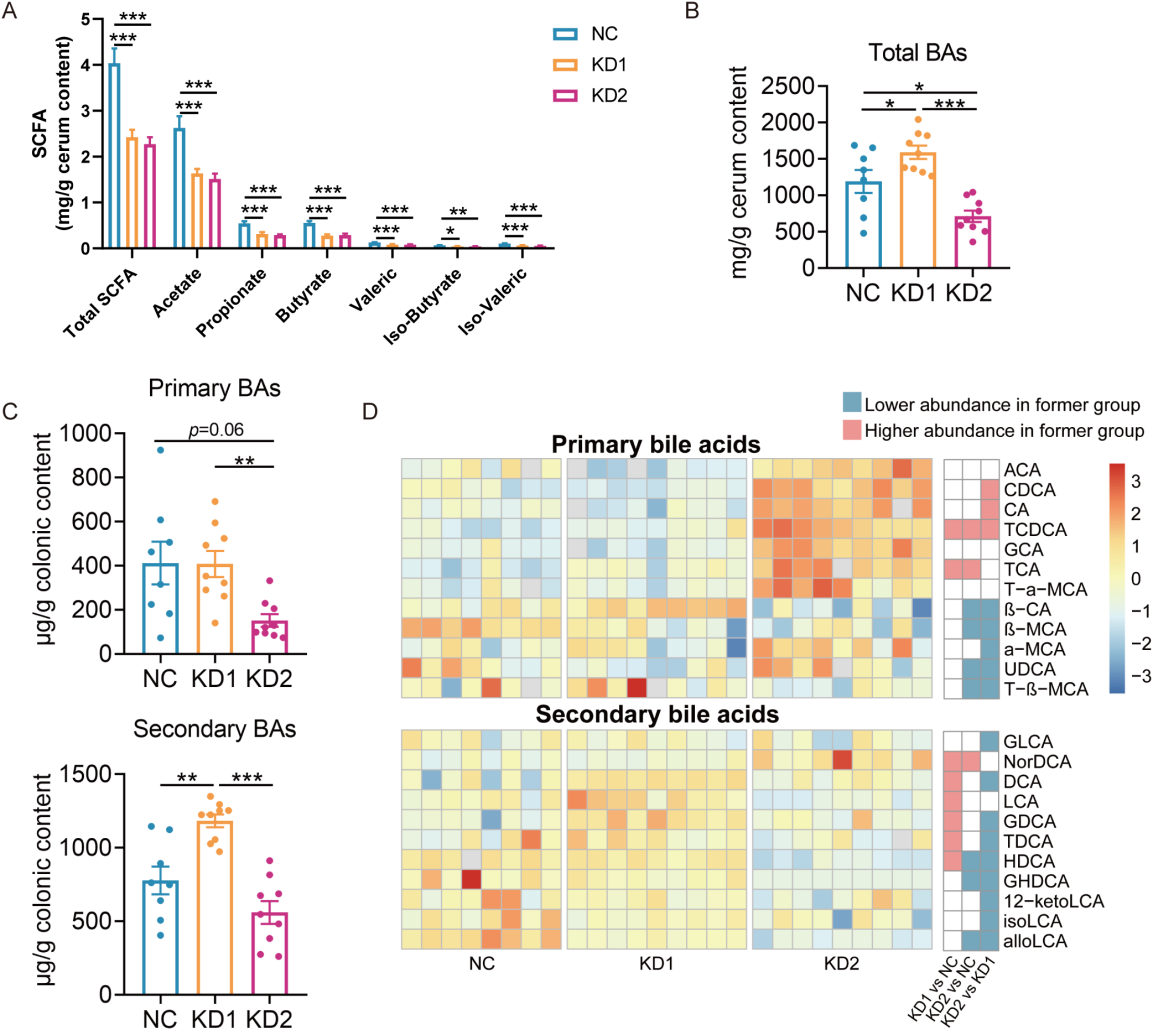
Changes of SCFA and bile acids in mice fed with two KDs. **(A)** Concentration of SCFAs (acetate, propionate, butyrate, iso-butyrate, valeric and iso-valeric) in cecal content of mice. **(B)** Concentrations of total bile acids in colon content of mice **(C)** Primary and secondary bile acid content. **(D)** The difference of bile acid content between groups through pairwise comparison of groups analyzed by the two-tailed tested using Mann-witney test, *p* < 0.05 was considered significant, and the blue indicated that the former was significantly lower than the latter, red indicated that the former was significantly higher than the latter and white indicated that there was no significant difference between the two groups. Data were presented as mean ± SEM and analyzed using one-way ANOVA test, followed by Tukey’s multiple comparisons test among NC, KD1 and KD2 groups, *p < 0.05, **p < 0.01, ***p < 0.001. In **(A)**, n = 10 for each group. In **(B, C)**, n = 8 for NC, n = 9 for KD1 and KD2.

Short-chain fatty acids (SCFAs) are the products of dietary fiber fermentation by gut microbiota, influenced by diet and the composition of gut microbiota, and closely related to the glucose and lipid metabolism of the host (24). The contents of each short-chain fatty acid in KD1 and KD2 mice significantly decreased compared to NC mice, with no significant difference in the content of these SCFAs between the KD1 and KD2 groups (**Figure 4A**). We conducted Spearman correlation analysis between the levels of each short-chain fatty acids and 58 ASVs selected based on RDA analysis associated with carbohydrate intake. We found that 18 ASVs showed significant positive correlations with at least one short-chain fatty acid (**Supplementary Figure S3**), with certain bacteria within the genera *Parabacteroides*, *Blautia*, and *Butyricimonas* reported to have the ability to produce SCFAs. Among these 18 ASVs, the relative abundance of 16 ASVs was significantly lower in both KD1 and KD2 groups compared to the NC group, while ASV 25 and ASV 26 demonstrated significantly lower relative abundances exclusively in the KD2 group compared to the NC group. These ASVs, responsive to reduced carbohydrate intake in the KD diet, may have the capacity to produce SCFAs, potentially resulting in decreased SCFA levels in the mouse gut and subsequent disruption of carbohydrate metabolism. Bile acids (BAs), crucial for facilitating the decomposition and absorption of dietary lipids, are influenced by the composition and content of dietary fat (25). The gut microbiota metabolizes primary bile acids into secondary bile acids, altering their composition and concentration, thus affecting host glucose metabolism (26). To explore whether gut microbiota participate in the disorder of glucose metabolism caused by the ketogenic diet by affecting bile acid metabolism, we measured the colonic bile acid profile. In comparison to the NC group, the KD1 group exhibited a significant increase in total bile acid content, whereas the KD2 group showed a significant decrease (**Figure 4B**). Additionally, KD2 significantly decreased the content of primary BAs, while KD1 significantly increased secondary BAs compared to the NC group (**Figures 4C, D**). Furthermore, we predicted the abundance of genes related to bile acid metabolism pathway of gut microbiota under two types of ketogenic diets based on PICRUSt2. We found that the relative abundance of *cbh*, encoding cholylglycine hydrolase, was markedly lower in the KD2 group compared to both the NC and KD1 groups. Conversely, the abundance of *hbhA*, encoding 7-α-hydroxysteroid dehydrogenase, was notably higher in the KD1 group in compared to both the NC and KD2 groups (**Supplementary Figure S4**). Taken together, these results suggest that KD intervention changes the bacteria related metabolites in the gut.

### 3.3 KDs induced lipid accumulation in ABX mice, but not glucose intolerance

To find out whether gut microbiota played roles in the mechanisms of KDs induced glucose and lipid metabolism disorders, we treated C57BL/6 mice with a cocktail of antibiotics for 4 weeks, which depleted the gut microbiota by more than 99% (**Supplementary Figure S5**). Then the mice were divided into three groups and administered their respective diets. After two weeks KD intervention, the blood ketone levels in ABX + KD1 and ABX + KD2 mice increased significantly compared to those in ABX + NC mice (**Supplementary Figure S6A**).

**Figure 5:**
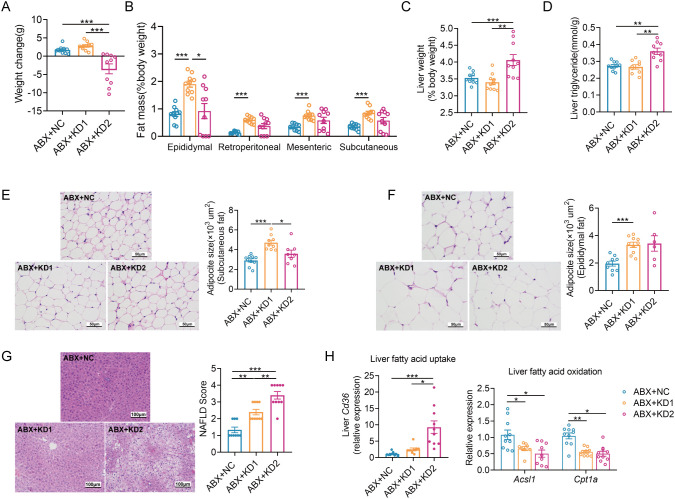
Two KDs also induced lipid accumulation in ABX mice. **(A)** Changes of body weight. **(B)**Weight coefficient of adipose tissue (epididymal fat, perirenal fat, mesenteric fat and subcutaneous fat, %body weight). **(C)** Liver weight. **(D)** Concentration of liver TG. **(E, F)** Representative H&E-stained histological sections of subcutaneous fat and epididymal fat (scale: 50 µm) and calculated mean cell area of adipocytes. **(G)** Representative H&E-stained sections of liver (scale: 100 µm) and calculated histologic score (NAFLD Score). **(H)** Relative mRNA expression of genes involved in fatty acid uptake and oxidation in liver. Data were presented as mean ± SEM and analyzed using one-way ANOVA test, followed by Tukey’s multiple comparisons test. ROUT (Q = 1%) was used in each group to eliminate outliers. *p < 0.05, **p < 0.01, ***p < 0.001. n = 10 for each group (except **D**). Since the 3 mice in ABX + KD2 group are too thin to collect subcutaneous adipose tissues, the analysis results of 7 mice were shown in **(D)**.

Similar to conventional mice, the ABX-treated mice had a significant reduction in body weight with KD2 intervention (**Figure 5A**, **Supplementary Figure S6B**). Moreover, white adipose tissue weight increased significantly only in the ABX + KD1 group, while liver weight and TG content increased significantly exclusively in the ABX + KD2 group, consistent with conventional mice (**Figures 5B–D**). Similarly, the area of epididymal and subcutaneous adipocytes increased significantly only in the ABX + KD1 group (**Figures 5E, F**), while NAFLD Scores were markedly elevated in both the ABX + KD1 and ABX + KD2 groups compared to the ABX + NC group (**Figure 5G**). Furthermore, the mRNA expression of the fatty acid uptake gene *Cd36* in the liver was also significantly up-regulated in the ABX + KD2 group, while the mRNA expression of fatty acid β-oxidation-related genes *Acsl1* and *Cpt1a* was significantly down-regulated in both the ABX + KD1 and ABX + KD2 groups (**Figure 5H**). Overall, both KD1 and KD2 caused lipid accumulation and fatty acid metabolism disorder under the gut microbiota-depleted condition (**Supplementary Figure S6**). These findings were consistent with the lipid metabolism phenotype induced by KDs in conventional mice (**Figure 1**), suggesting that the lipid metabolism abnormalities induced by KD in mice are independent of gut microbiota.

**Figure 6:**
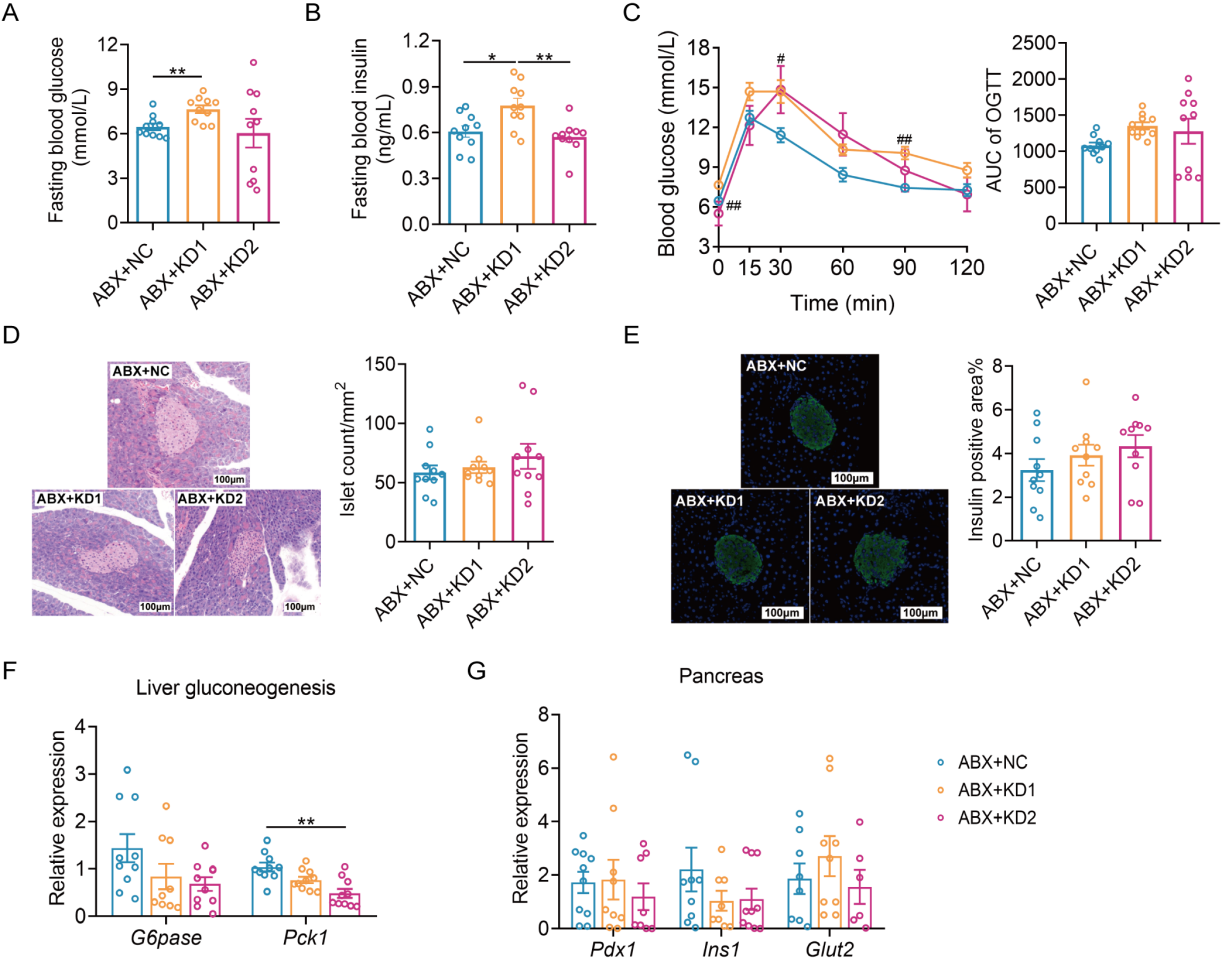
Neither KD1 nor KD2 induced glucose intolerance in ABX mice. **(A)** Fasting blood glucose. **(B)** Fasting blood insulin. **(C)**Blood glucose curve and area under the curve (AUC) in the oral glucose tolerance test (OGTT). **(D)** Representative pancreatic tissue H&E staining and calculated islet number and islet area. **(E)** Representative insulin immunofluorescence-stained (green) histological sections of pancreas (scale bar = 100 μm and calculated mean insulin-positive area. **(F)** Relative mRNA expression of genes involved in liver gluconeogenesis. **(G)** Relative mRNA expression of genes of pancreas. All data were presented as mean ± SEM and analyzed using one-way ANOVA test, followed by Tukey’s multiple comparisons test among NC, KD1 and KD2 groups. In **(C)**, # and * represent the results of Tukey’s multiple comparison test between KD1 group and NC group, KD2 group and NC group respectively. ROUT (Q = 1%) was used in each group to eliminate outliers. *p < 0.05, **p < 0.01, ^#^ *p* < 0.05, ^##^ *p* < 0.01, n = 10 for each group.

Then we evaluated the effects of the two types of KD on glucose metabolism in ABX mice. Fasting blood glucose and insulin levels were notably elevated in the ABX + KD1 group compared to the ABX + NC group, while no significant difference was observed between the ABX + KD2 group and the ABX + NC group, differing from conventional mice (**Figures 6A, B**). Moreover, glucose levels at 30 and 60 minutes during an OGTT in the ABX + KD1 group were significantly higher than those in the ABX + NC group after oral glucose administration, but the glucose AUC did not show a statistical difference (**Figure 6C**). Meanwhile, there was no significant difference in blood glucose levels at each time point or in the 2-hour glucose AUC during the OGTT between the ABX + KD2 group and the ABX + NC group (**Figure 6C**). The above results showed that the glucose metabolic phenotypes in ABX mice fed with KDs were different from those in conventional mice (**Figures 2A–C**, **6A–C**).

More interestingly, we observed no significant differences in the insulin positive area or the average number of pancreatic islets among the ABX mice fed the three diets (**Figures 6D, E**). Furthermore, there were no significant differences in the mRNA expression of gluconeogenesis-related gene *G6pase* among the three groups. Only the mRNA expression of *Pck1* was down-regulated in the ABX + KD2 group (**Figure 6F**), and there were no significant differences in the mRNA expression levels of *Pdx1*, *Ins1*, and *Glut2*—genes related to insulin synthesis and release in the pancreas—among the three groups (**Figure 6G**). Taken together, these results indicate that ketogenic diets resulted in glucose metabolism disorders in conventional mice but not in microbiota-depleted mice (**Figures 2**, **6**), suggesting a dependence of ketogenic diet-induced glucose metabolism disorders in mice on gut microbiota.

## 4 Discussion

Overall, the results of the current work showed that two types of KDs used in clinical studies and animal experiments, caused the disorders of glucose and lipid metabolism in mice. Furthermore, the gut microbiota changed by ketogenic diets involved in glucose intolerance rather than lipid accumulation.

Consistent with previous studies (15, 16), we observed that two types of KDs induced abnormal glucose and lipid metabolism in mice, and both altered the gut microbiota structure. In current research, we found that the gut microbiota mediated glucose metabolism disorders induced by KDs, while KD-induced lipid metabolism disruptions were independent of changes in gut microbiota. Specifically, due to the high fat content in the KD, it affects the expression of genes related to host fatty acid uptake and oxidation, leading to dysregulation of lipid metabolism. Consistently, a previous study has reported that one potential mechanism by which ketogenic diets affect lipid metabolism is by reducing fat synthesis and increasing fat oxidation (27). Interestingly, KD1 resulted in increased peripheral fat rather than hepatic fat accumulation. Concurrently, peripheral fats such as epididymal fat showed increased expression of genes related to triglyceride synthesis, decreased expression of genes related to fatty acid oxidation and lipolysis. Additionally, hepatic fatty acid oxidation decreased. Given the ketogenic diet’s inherently high fat content, this difference may be due to imbalances in triglyceride synthesis, fatty acid oxidation, and lipolysis resulting from nutritional imbalances in the diet. To summarize, the ketogenic diet directly impacts host metabolism, causing abnormalities in host lipid metabolism. In addition, we observed decreased gut microbiota diversity and lower levels of gut SCFAs in KD-fed mice. Furthermore, there was a reduction in the abundance of certain common SCFA-producing bacteria, including *Blautia* (ASV 122, ASV 138, ASV 227) and *Alistipes* (ASV 16) (28). Studies have shown that reduced SCFAs production due to gut microbiota dysbiosis may impair intestinal gluconeogenesis and glucose homeostasis, contributing to glucose metabolism disorders (29). Specifically, SCFAs have been demonstrated to stimulate pancreatic insulin secretion and improve glucose tolerance by acting on G protein-coupled receptors (GPCRs) expressed on pancreatic beta cells (29). It is speculated that the low carbohydrate content in the ketogenic diet leads to decreased levels of SCFAs, which are metabolites produced by the gut microbiota using carbohydrates as substrates, resulting in impaired glucose metabolism in mice. Additionally, both KDs significantly altered the bile acid profile. Research shows that disruption of the gut microbiota can lead to bile acid profile dysregulation, which might contribute to the development of chronic inflammatory diseases such as type 2 diabetes (T2D) and colon cancer (26). In summary, gut microbiota dysbiosis induced by the low-carbohydrate, high-fat ketogenic diet, leading to decreased SCFA levels and alterations in bile acid profile, subsequently resulting in insulin resistance and reduced pancreatic insulin secretion, may represent one of the mechanisms underlying glucose metabolism disorders caused by ketogenic diets.

Previous research suggests that the ketogenic diet effectively reduces fasting blood glucose levels, making it a viable option for managing type 2 diabetes (30, 31). The latest research shows that feeding mice with a ketogenic diet significantly reduces body weight and fasting blood glucose levels (32). In our study, although KD2 lowered fasting blood glucose levels, it still led to impaired glucose tolerance, accompanied by a decrease in insulin secretion. The decrease in the expression levels of *G6pase* and *Pck1* genes indicates a correlation between the decreased in fasting blood glucose and reduced hepatic gluconeogenesis, consistent with the reliance of mice on ketones and fatty acids as energy substrates under the KD. A study indicated that mice with reduced *Pck1* expression develop insulin resistance, hypoglycemia, and hepatic steatosis (33). Therefore, it is hypothesized that the elevated liver triglycerides caused by KD2 may be associated with the high-fat, low-carbohydrate dietary pattern. In summary, the decrease in fasting blood glucose caused by the ketogenic diet does not indicate improved metabolism but rather denotes instability and abnormal glucose processing. Interventions using a ketogenic diet in T2D patients may exacerbate glucose metabolism issues. Furthermore, consistent with the weight loss effects of the ketogenic diet, KD2 resulted in decreased body weight in mice, but this weight loss comes at the expense of hepatic fat accumulation and lipid metabolism imbalance. Prolonged exposure may lead to fatty liver disease and non-alcoholic fatty liver disease. Consistent with our study, previous studies have reported that KD induces glucose intolerance, insulin resistance and hepatic lipid accumulation in mice (16, 17, 34, 35). Recently, research has revealed that just 21 days on a ketogenic diet can induce cellular senescence in various organs such as the heart, kidneys, liver, and brain in mice, highlighting the health risks associated with ketogenic diets (36). In summary, our study underscores the detrimental effects of short-term ketogenic diets on glucose and lipid metabolism. Ketogenic diets should not be considered metabolically healthy, and the associated health risks should be fully considered when using them to treat diseases or facilitate weight loss.

In both research and real life, ketogenic diet formulations vary, which may explain the differing effects observed across studies. The two types of ketogenic diets we employed shared the same nutritional sources but differed in their proportions. Notably, the lower-protein KD2 induced significant hepatic fat accumulation in mice. Previous studies have reported that the KDs with an appropriate amount of protein (caloric content of around 20%) can induce obesity and insulin resistance (16, 37, 38), while those with protein restriction (caloric content of less than 10%) may lead to hepatic lipid accumulation in mice (17, 39, 40). Tricò, D et al. reported that protein restriction regulates fatty acid uptake and metabolism by reducing the expression level of PPARα in the liver (41). Conversely, research has demonstrated that a high-protein diet can promote metabolic health by reducing both liver and overall body fat levels (41). This may explain why an animal KD causes lipid accumulation in the liver due to protein restriction. Additionally, KD does not always lead to weight loss. Studies have shown that the weight loss effect of KD is probably related to ketones, which can reduce appetite and increase energy consumption (42, 43). Fats are predominantly ketogenic, carbohydrates are almost anti-ketogenic, and protein is both ketogenic and antiketogenic. Therefore, only when the ketogenic ratio calculated from the mass of three macronutrients is higher than 1.7 can it cause fat decomposition and weight loss (44, 45). In current research, KD1 caused weight gain and accumulation of subcutaneous and visceral fat. Compared to KD1 mice, the blood ketone level was higher in KD2 mice, indicating a weight-loss effect. Importantly, the two types of ketogenic diets shaped entirely distinct gut microbiota structures, particularly affecting key ASVs responding to protein and fat. Compared to NC diet and KD1, the lower-protein KD2 downregulated the abundance of Muribaculaceae while upregulating taxa such as Lachnospiraceae, Ruminococcaceae, and Desulfovibrionaceae. A study shows that compared to a standard protein diet, a high-protein diet leads to an increase in some disease-associated bacteria (such as *Escherichia/Shigella*, *Enterococcus*, and *Streptococcus*) and a decrease in beneficial bacteria (such as *Ruminococcus*, *Akkermansia*, and *Faecalibacterium prausnitzii*), while a low protein diet leads to a higher abundance of Desulfovibrionaceae (positively correlated with inflammation) (46). Thus, a higher or lower protein intake could have a negative impact on gut microbiota and, subsequently, on health, and there appears to be an optimum protein intake required for normal gut health. In summary, although the ketogenic diet is defined as a diet high in fat, low in carbohydrate, and moderate in protein, in fact, subtle differences in specific nutrients can lead to variations in the effects on host metabolic and its gut microbiota. In future studies, the design of ketogenic diet formulations needs to be more precise, considering the specific nutrient content and potential synergies between nutrients, as well as their effects on the gut microbiota.

In conclusion, our study reveals that the glucose metabolism abnormalities, rather than lipid metabolism abnormalities, induced by commonly used KDs in clinical studies and animal experiments depend on the gut microbiota, indicating a direction for further elucidating the mechanisms underlying ketogenic diet-induced metabolic disturbances in both glucose and lipid metabolism. It should be noted that a ketogenic diet is a highly unbalanced eating pattern that not only directly affects host metabolism but also indirectly impacts host health through gut microbiota. When utilized for long-term treatment of chronic diseases, it’s crucial to carefully design nutrient ratios to mitigate health risks and promote optimal metabolic health for both the host and its microbiota.

## Data Availability

The datasets presented in this study can be found in online repositories. The names of the repository/repositories and accession number(s) can be found in the article/**Supplementary Material**.
