# Supplementary material for: The gut microbiota changed by ketogenic diets contribute to glucose intolerance rather than lipid accumulation
Source: Front Endocrinol (Lausanne). 2024 Sep 27;15:1446287. doi: 10.3389/fendo.2024.1446287 (PMC11466860; doi:10.3389/fendo.2024.1446287)
Supplement: Supplementary file 1 [file DataSheet1.docx]

Supplementary Material

# Supplementary Figures and Tables

## Supplementary Figure 1

**
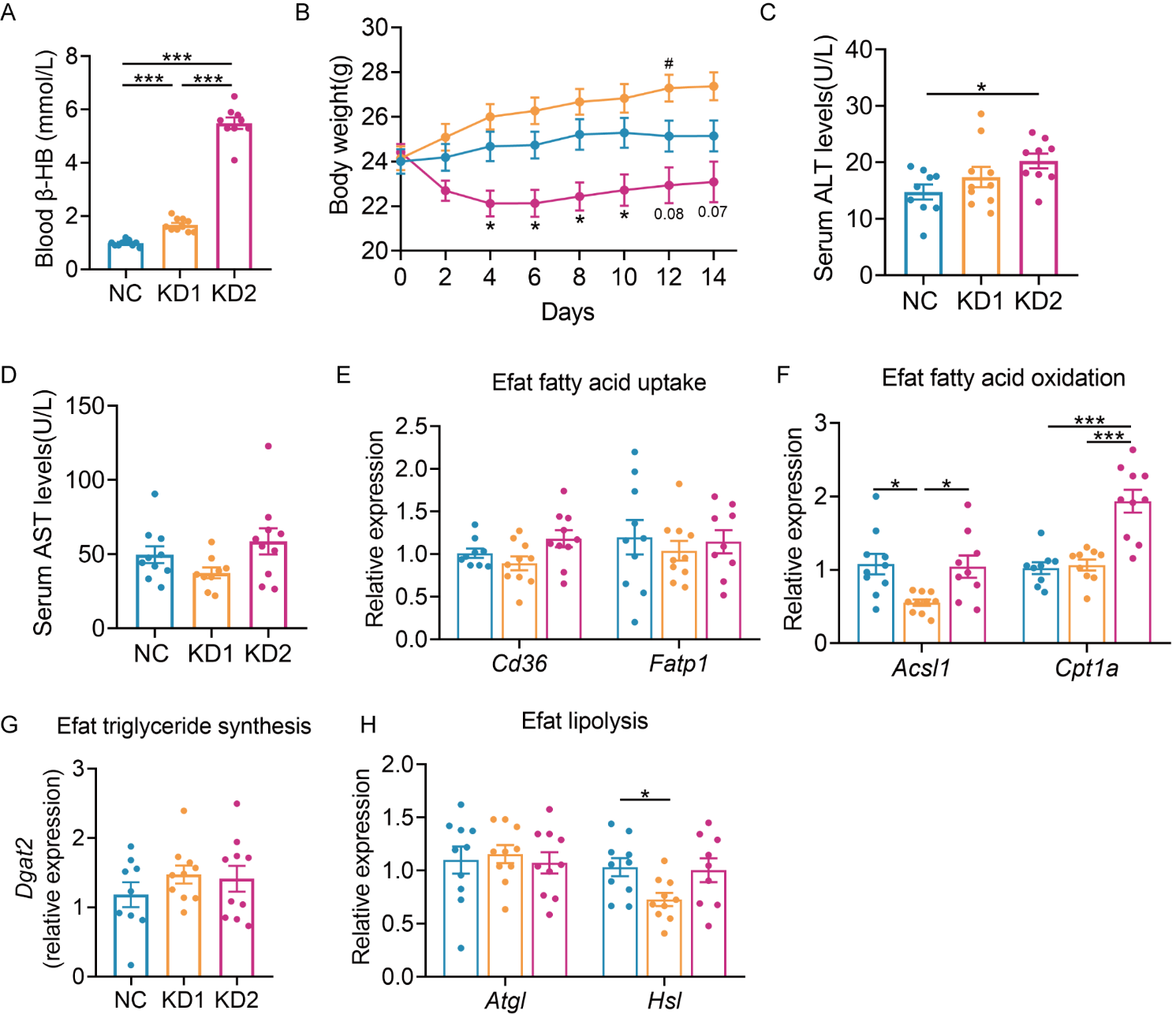
Supplementary Figure 1.** **KD induced lipid metabolism disorders in SPF mice.** (A) Fasting blood ketone. (B) Changes of body weight. (C) Concentration of ALT in serum. (D) Concentration of AST in serum. (E-H) Relative mRNA expression of genes involved in fatty acid uptake (*Cd36*, *Fatp1*) and oxidation (*Acsl1*, *Cpt1a*), TG synthesis (*Dgat2*) and lipolysis (*Atgl, Hsl*) in epididymal fat. Data were presented as mean ± SEM and analyzed using one-way ANOVA test, followed by Tukey’s multiple comparisons test. ROUT (Q = 1%) was used in each group to eliminate outliers. **p* ＜ 0.05, ***p* ＜ 0.01, ****p* ＜ 0.001. n = 10 for each group.

##
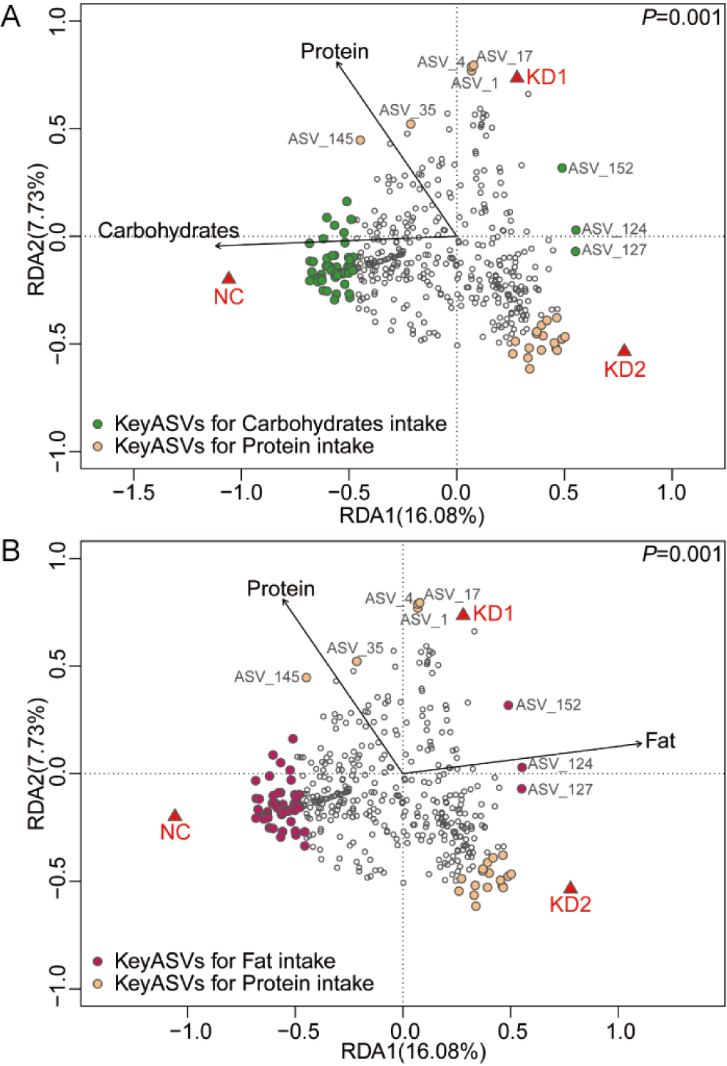
Supplementary Figure 2

**Supplementary Figure 2.** **The ASVs responding to different nutrient intake under KD.** RDA biplot with carbohydrate intake and protein intake (A), fat intake and protein intake as environmental factors (explanatory variables) and relative abundance of ASVs as response variables. Arrows represent environmental factors, which explained 23.81% of the variation in microbiota structure. The red triangles represent the different experimental groups. Solid points in different colors represent key ASVs in response to different nutrient intakes. The *p* value was analyzed by Mantel test to analyze the significance of environmental factors on microbial structure.

## Supplementary Figure 3


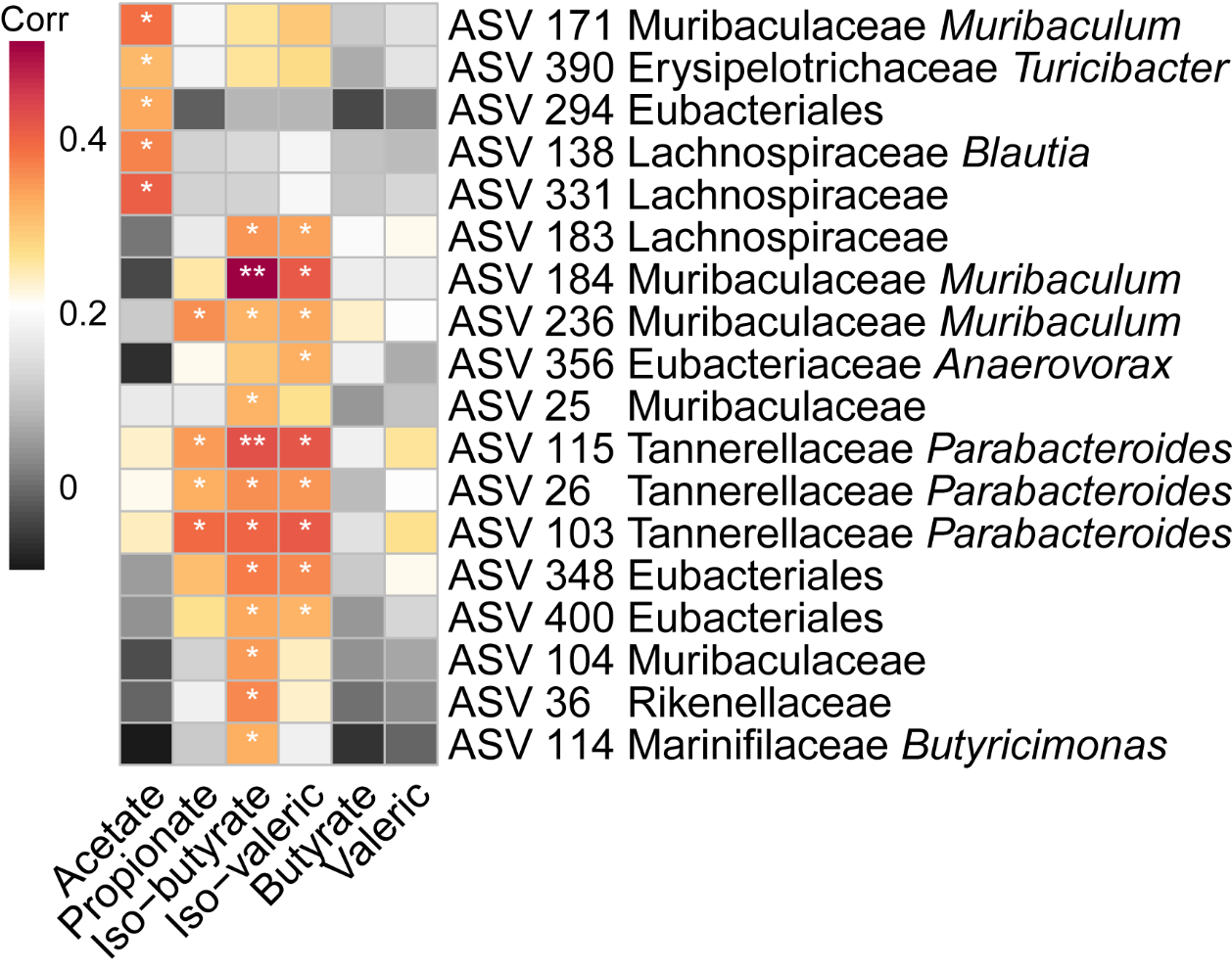
**Supplementary Figure 3.** **Key ASVs responding to carbohydrates/fat intake are associated with the concentrations of SCFAs.** Spearman correlations between the relative abundance of 18 ASVs and the concentration of SCFAs. Positive correlation (red), negative correlation (black). p values were FDR corrected, **p* ＜ 0.05, ***p* ＜ 0.01, ****p* ＜ 0.001. n = 10 for each group.

##
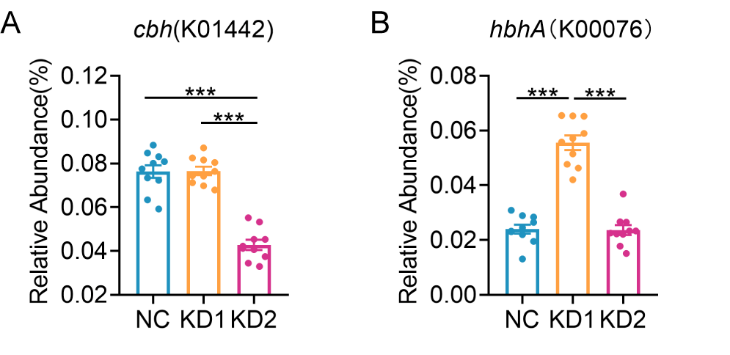
Supplementary Figure 4

**Supplementary Figure 4.** **Relative abundance of genes related to bile acid metabolism predicted by PICRUSt2.** (A) The relative abundance of bacterial choloylglycine hydrolase (*cbh*, K01442). (B) The relative abundance of bacterial 7-α-hydroxysteroid dehydrogenase (*hbhA,* K00076). Data were presented as mean ± SEM and analyzed using one-way ANOVA test, followed by Tukey’s multiple comparisons test. **p* ＜ 0.05, ***p* ＜ 0.01, ****p* ＜ 0.001. n = 9 for each group.

##
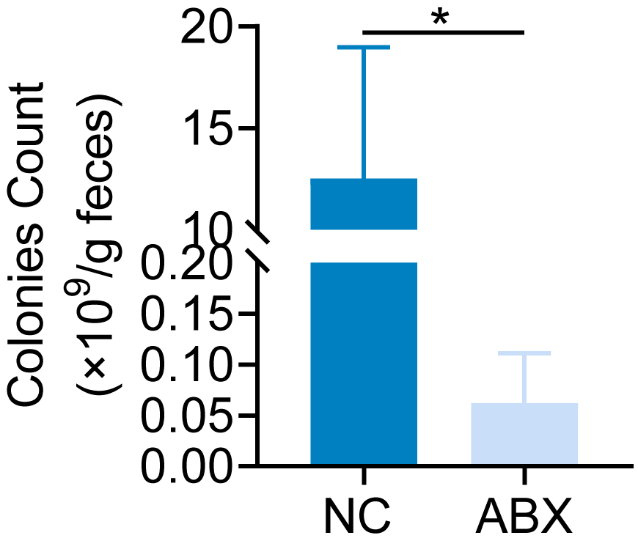
Supplementary Figure 5

**Supplementary Figure 5.** **The gut microbiota had been depleted by more than 99% in the mice with a cocktail of antibiotics for 4 weeks.** The fecal samples from a cage of mice were diluted and spread on the surface of BHI and LB agar medium plates, and the BHI plates were placed in the anaerobic workstation (80% N_2_, 10% CO_2,_ and 10% H_2_ at 37°C), the LB plates were placed in the 37°C incubator with air. After 48 hours, the colonies count was performed. The colonies count was represented as the sum of the colonies from BHI and LB agar medium plates. NC group, n = 3; ABX group, n = 3. NC, the control mice treated with pure drinking water; ABX, the antibiotic-cocktail treated mice with pure water. Data were presented as mean ± SEM, and Mann-Whitney test (one-tailed) was used to analyze differences between NC vs. ABX. **p* ＜ 0.05.

## Supplementary Figure 6

**Supplementary Figure
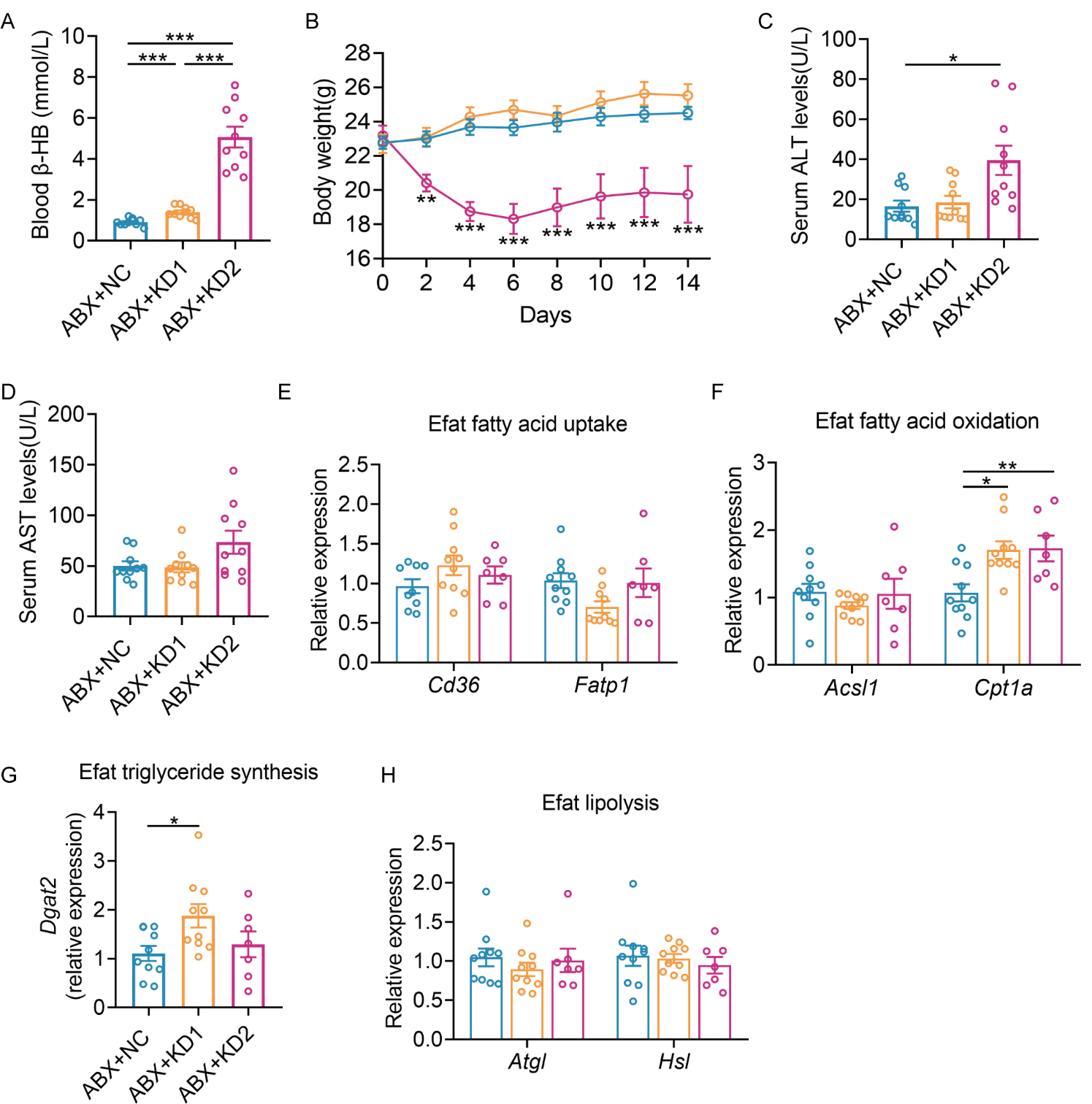
6.** **Effects of two kind of KD on adipose tissue lipid metabolism in ABX mice.** (A) Fasting blood ketone. (B) Changes of body weight. (C) Concentration of ALT in serum. (D) Concentration of AST in serum. (E-H) Relative mRNA expression of genes involved in fatty acid uptake (*Cd36*, *Fatp1*) and oxidation (*Acsl1*, *Cpt1a*), TG synthesis (*Dgat2*) and lipolysis (*Atgl, Hsl*) in epididymal fat.Data were presented as mean ± SEM and analyzed using one-way ANOVA test, followed by Tukey’s multiple comparisons test. ROUT (Q = 1%) was used in each group to eliminate outliers. **p* ＜ 0.05, ***p* ＜ 0.01, ****p* ＜ 0.001. Since the 3 mice in ABX + KD2 group are too thin to collect subcutaneous adipose tissues, the analysis results of 7 mice were shown in Figure (E-H). n = 10 for each group in other plots.

## Supplementary Tables

**Supplementary Table 1. Components of diets**

| Ingredient (g/kg) | NC | KD1 | KD2 |
| --- | --- | --- | --- |
| Casein | 200.00 | 287.19 | 121.00 |
| DL-Methionine | 3.00 | 4.31 | 1.56 |
| Corn Starch | 389.00 | 71.23 | 0.00 |
| Maltodextrin | 100.00 | 18.31 | 0.00 |
| Sucrose | 150.00 | 27.47 | 0.00 |
| Corn oil | 8.75 | 57.85 | 86.20 |
| Vegetable Shortening, hydrogenated (Crisco) | 61.25 | 404.49 | 605 |
| Cellulose | 50.00 | 67.42 | 112.95 |
| Mineral Mix, Ca-P Deficient (79055) | 13.37 | 19.40 | 23.80 |
| Vitamin Mix, Teklad (40060) | 10.00 | 14.50 | 17.80 |
| Calcium Phosphate, dibasic | 7.50 | 16.39 | 24.30 |
| Calcium Carbonate | 6.85 | 5.87 | 4.40 |
| Magnesium Oxide | 0.20 | 0.29 | 0.35 |
| Carbohydrate (kcal) | 65.6% | 8.8% | 0.4% |
| Protein (kcal) | 18.0% | 17.9% | 6.4% |
| Fat (kcal) | 16.3% | 73.4% | 93.2% |
| Energy Density (kcal/g) | 3.9 | 5.7 | 6.7 |

**Supplementary Table 2. Daily energy intake of nutrients in each group per mice**

| Group | Carbohydrates(kcal/mice) | Fat(kcal/mice) | Protein(kcal/mice) |
| --- | --- | --- | --- |
| NC | 1.24 | 0.307 | 0.339 |
| KD1 | 0.208 | 1.74 | 0.424 |
| KD2 | 0.009 | 2.039 | 0.140 |
